# Retraction: Single walled carbon nanotubes reinforced mineralized hydroxyapatite composite coatings on titanium for improved biocompatible implant applications

**DOI:** 10.1039/d3ra90024j

**Published:** 2023-03-28

**Authors:** D. Gopi, E. Shinyjoy, A. Karthika, S. Nithiya, L. Kavitha, D. Rajeswari, Tingting Tang

**Affiliations:** a Department of Chemistry, Periyar University Salem 636011 Tamilnadu India dhanaraj_gopi@yahoo.com +91 427 2345124 +91 427 2345766; b Centre for Nanoscience and Nanotechnology, Periyar University Salem 636011 Tamilnadu India; c Department of Physics, School of Basic and Applied Sciences, Central University of Tamilnadu Thiruvarur 610 101 Tamilnadu India; d Shanghai Key Laboratory of Orthopaedic Implants, Department of Orthopedic Surgery, Shanghai Ninth People’s Hospital, Shanghai Jiaotong University School of Medicine 639 Zhizaoju Road Shanghai 20011 P. R. China

## Abstract

Retraction of ‘Single walled carbon nanotubes reinforced mineralized hydroxyapatite composite coatings on titanium for improved biocompatible implant applications’ by D. Gopi *et al.*, *RSC Adv.*, 2015, **5**, 36766–36778, https://doi.org/10.1039/C5RA04382D.

The Royal Society of Chemistry, with the agreement of the named authors, hereby wholly retracts this *RSC Advances* article due to concerns with the reliability of the data in the published article.

There are unexpected similarities in the XRD patterns presented in Fig. 2a–c in the 22–24° and 28–56° regions.

The left-hand side of the cross-sectional SEM image in Fig. 3d has been duplicated in other publications,^1–3^ whereas the right-hand section of the image in Fig. 3d has been duplicated in another article.^4^

The authors informed the Editor that the characterization of the original samples was outsourced, and they do not have the original raw data for the published results.

Given the significance of the concerns about the validity of the data, and the lack of raw data, the findings presented in this paper are not reliable.

S. Nithiya and Tingting Tang were contacted but did not respond.

Signed: D. Gopi, E. Shinyjoy, A. Karthika, L. Kavitha and D. Rajeswari

Date: 16th March 2023

Retraction endorsed by Laura Fisher, Executive Editor, *RSC Advances*

## Supplementary Material

## References

[cit1] Chozhanathmisra M., Ramya S., Kavitha L., Gopi D. (2016). Colloids Surf., A.

[cit2] Gopi D., Sherif E.-S. M., Rajeswari D., Kavitha L., Pramod R., Dwivedi J., Polaki S. R. (2014). J. Alloys Compd..

[cit3] Gopi D., Arumugam K., Nithiya S., Kavitha L. (2014). Mater. Chem. Phys..

[cit4] Gopi D., Karthika A., Rajeswari D., Kavitha L., Pramod R., Dwivedi J. (2014). RSC Adv..

